# The ethics of diplomatic criticism: The Responsibility to Protect, Just War Theory and Presumptive Last Resort

**DOI:** 10.1177/1354066115572491

**Published:** 2015-03-31

**Authors:** James Pattison

**Affiliations:** University of Manchester, UK

**Keywords:** Diplomatic criticism, duty to criticise, ethics, hypocrisy, Just War Theory, Responsibility to Protect

## Abstract

This article presents the ethical case for diplomatic criticism as a response to mass atrocities and serious external aggression. It argues, in short, that states have a moral duty to criticise the offending parties. More specifically, it argues that diplomatic criticism is often a plausible and preferable alternative to other means of addressing serious external aggression and mass atrocities (such as war, economic sanctions and other diplomatic measures). It also argues that diplomatic criticism is often preferable to doing nothing, and that even if other means are undertaken, states should engage in diplomatic criticism as well. There are two subsidiary aims of the article. The first is to reject some of the worries surrounding international hypocrisy — I aim to show that even hypocritical diplomatic criticism may be obligatory. The second is to highlight the impact on Just War Theory of considering in more detail the ethical issues raised by the alternatives to war, such as diplomatic criticism, and, more specifically, to present a new account of the last resort principle, which I call ‘Presumptive Last Resort’.

## Introduction

How should states and supranational bodies respond when faced with a state that threatens another political community or initiates mass atrocities against its own population? The most frequently discussed options in the literatures on collective self-defence and the Responsibility to Protect (R2P) doctrine are war and military intervention. Almost all accounts of Just War Theory and R2P assert that coercive military action should be avoided as far as possible: war and intervention should be the last resort. Stricter accounts of the last resort principle take the ‘last’ element literally, requiring that war or intervention should be pursued only after all the alternatives have been tried. More permissive, non-literal accounts of last resort assert that war or intervention should be pursued only if the other options are unlikely to succeed or if war or intervention would lead to the least amount of harm.^1^ Notwithstanding, the notion that alternatives to war should be pursued is one of the least controversial elements of Just War Theory and R2P. Yet, if war is to be avoided, what should be done instead? Economic sanctions are a notable alternative, but, as has been well documented, they can cause devastation on a similar scale to that of war, such as the economic sanctions against Iraq in the 1990s (Gordon, 2010), and their effectiveness is highly contested.^2^

The alternative viewed perhaps most favourably is diplomacy. Diplomacy is widely cited in the discussions of last resort in Just War Theory and R2P as a desirable alternative to war and intervention that should be pursued first.^3^ However, almost all accounts of last resort do not consider in detail what diplomacy would or should involve. Nor do they explore the ethical issues surrounding diplomacy as a means of addressing external aggression and mass atrocities. In a similar vein, pacifists often call for diplomacy as an alternative to war but do not consider the moral issues for and against this option (beyond the repudiation of war). For its part, the field of diplomatic studies is largely disparate and multidisciplinary, involving disciplines such as communication studies, foreign policy analysis, International Relations (IR), public relations and sociology. However, within this field, there is very little written on diplomacy from a normative perspective.^4^

Accordingly, in this article, I will consider the ethics of diplomacy. I will focus, in particular, on the use of diplomatic criticism as a response to serious external aggression and mass atrocities. The main aim of the article is to present the case for diplomatic criticism as a response to such situations. To that extent, in regard to responding to mass atrocities (genocide, war crimes, ethnic cleansing and crimes against humanity), I will defend diplomatic criticism as a central means of helping to fulfil the R2P. Overall, I will argue that states have a prima facie moral duty to engage in international criticism.

More specifically, I will argue that diplomatic criticism is not simply doing nothing and standing by (as is often perceived in the public debate), but, rather, (1) is often a plausible and preferable *alternative* to other means of addressing mass atrocities and serious external aggression, such as war, economic sanctions and other diplomatic measures (dialogue, mediation and the cutting and downgrading of political ties). In addition, I will argue (2) that diplomatic criticism is often a plausible and preferable *alternative* to doing nothing; I will argue that staying silent in the face of serious external aggression or mass atrocities is morally problematic. That is to say, states are often morally required to criticise the offenders, in contrast to the numerous cases where they have stayed silent in the face of human rights abuses (cases include: South African failure to criticise Robert Mugabe’s Zimbabwe; Australian failure in the 2013 Commonwealth Meeting to criticise the abuses of human rights by the Sri Lankan government; the failure of the UK (under Margaret Thatcher) to criticise General Pinochet’s human rights abuses in Chile; and British and American failure to criticise human rights abuses in Uzbekistan).^5^ I will also argue that (3) diplomatic criticism should often be undertaken *in addition* to other ways of addressing serious external aggression and mass atrocities. Thus, I will argue that states should often register diplomatic criticism of offenders both when they undertake other measures *and* when they do not (e.g. because military intervention would do more harm than good).

My argument will proceed as follows. In the second section, I will present the case for the duty to engage in international criticism. I will suggest that this duty stems from three bases: (i) efficacy at addressing the situation; (ii) contribution to global norms; and (iii) punishment. In the third section, I will defend diplomatic criticism as favourable to war and economic sanctions, as well as to three other diplomatic measures. In the fourth section, I will consider (and largely, if not fully, repudiate) two leading objections to the duty to engage in international criticism. The first — the ‘Demandingness Objection’ — is that the duty to criticise is far too demanding. The second — the ‘Hypocrisy Objection’ — is that the duty to criticise is highly circumscribed since states are not entitled to criticise others when they are being hypocritical. I will then (in the fifth section) delineate the import of the argument for Just War Theory and, in doing so, present a new account of the last resort principle, which I call ‘Presumptive Last Resort’.

Before beginning, I should make clear the scope of the article. I am concerned with a particular form of political sanctions — diplomatic criticism or statist ‘naming and shaming’, which is the identification and public criticism of offending agents by states. In general, political sanctions can take the form of ‘coercive diplomacy’ in that a state uses such sanctions — or the threat of them — to achieve certain political goals. However, I will not explore coercive diplomacy that concerns the threat of military or economic coercion. In addition, the focus of the article will be on states, although some of the argument may also apply to other agents, such as non-governmental organisations (NGOs) and the United Nations (UN). Furthermore, the focus will *primarily* be on serious external aggression (i.e. cases where defensive military force might be permissible) and internal mass atrocities (i.e. R2P cases). This is because these are the sorts of cases widely viewed as potentially providing just cause for war; I want to consider the case for diplomacy as an alternative in these cases. However, some of the analysis will also be relevant for (and concern) the issue of rebukes in other cases of wrongful behaviour by states, such as less severe forms of oppression of domestic populations and more minor forms of external interference.

## The duty to engage in international criticism

To establish that there is a duty to criticise, let us first consider the potential positive reasons for engaging in diplomatic criticism. These reasons help to provide the prima facie case for often pursuing diplomatic criticism over other options and, in doing so, help to defend the case for diplomatic criticism as a desirable option under the comparative conditions of Just War Theory, such as the last resort and necessity principles.

### Efficacy

The first is straightforward: although the efficacy of diplomatic criticism is often understated (e.g. it is viewed as simply doing nothing in response to aggression), naming and shaming may often be effective at addressing the situation. This is because, in short, people try to avoid criticism. More specifically, on constructivist accounts of IR, states are concerned with both the ‘logic of consequences’ — that is, maximising their fixed interests and preferences — and the ‘logic of appropriateness’ — conforming to expected behaviour determined by the prevailing norms of the international system (Finnemore, 1996; March and Olsen, 1998). In regard to the logic of appropriateness, states seek (perceived) legitimacy. When states are criticised, challenges are posed to their reputations and their international standing. If the criticism is widely upheld, the subsequent loss of legitimacy might be significant not only for the state’s interests (e.g. as it is barred from membership of international bodies and loses trade agreements), but also its self-identity (e.g. as its view of itself as a force for good in the international system is challenged).^6^

Consequently, if we accept that states care about the loss of legitimacy, and that diplomatic criticism is important for leading to such a loss, states will sometimes be keen to redress the situation at hand in response to diplomatic criticism, or under the threat of such criticism. The potential impact of such criticism is missed on materialist accounts of IR, which focus on material interests and on enforcement through only economic or military measures. Although certain states may prioritise material interests or be less worried about international criticism (e.g. because they are already international pariahs), challenges to international reputation do seem to lead to changes in certain states’ behaviour.^7^

Even when states are already engaged in other ways of addressing the crisis, such as war or economic sanctions, diplomatic criticism can help to put further pressure on the aggressor. Moreover, it can help in providing a clear public justification of war and economic sanctions. Indeed, if economic sanctions are to be justified in part symbolically, that is, by their expression of disapproval of the sanctioned party, and so to contribute generally to existing international prohibitions on aggression and other practices, as some defenders of economic sanctions argue (e.g. Damrosch, 1994; Pasternak, 2009), they need to be accompanied by an account of the reasons as to why the regime is to be sanctioned.

This argument about the efficacy of diplomatic criticism can be stated more precisely by drawing on Thomas Risse’s (2000) Habermasian account of communicative action. According to Risse, the logic of consequences (which, in effect, concerns the realm of rational choice, where agents pursue fixed interests and preferences) and the logic of appropriateness (which, as we have seen, concerns the import of social structures in influencing and constituting agents) should be supplemented with the ‘logic of argumentation’. The logic of argumentation concerns the role played by individual agents in influencing others’ interests or preferences. Actors will not always have fixed interests or preferences; these will be up for negotiation.

At times, all the actors engaged will be open to persuasion. This seems unlikely, however, for states reacting to serious external aggression or mass atrocities. Rather, *one* set of actors is more likely to be open to persuasion. That is to say, in the sorts of cases in which I am primarily interested in this article, the audience will often concern not simply the actors directly involved — the critic and the agent subject to the criticism — but also the international community more generally, including world public opinion. By providing an account of external aggression or mass atrocities and, in particular, by stating its empirical claims about the violations and asserting its normative claims about the wrongness of the violations, the critic is putting forward an argument that the subject of their criticism should amend their behaviour or face international opprobrium. The audience — the international community — is open to persuasion about the case at hand and will assess the case presented by the critic, as well as the responses of the alleged wrongdoers, such as that they are ‘fighting terrorists’. Although the situation may not match the requirements of the Habermasian Ideal Speech situation, the force of the better argument may still prevail.

Once persuaded, for instance, of the facts of the case, the relevant audience — actors in the international community — may repudiate the wrongdoer for failing to conform to the required normative standards of the international system. In order to fend off the opprobrium of the international community, the norm-violating government may make rhetorical concessions and cease denying the validity of or contesting human rights or related norms (Risse, 2000: 32). This may open them up to further criticism about their failure to live up to norms that they, in fact, endorse and may ultimately lead to a shift in the perceived preferences and interests of the norm violator, and a shift in behaviour, in order to be consistent (Risse calls this ‘self-entrapment’).

What goes on in such cases cannot be *solely* explained by the logic of consequences or the logic of appropriateness. This is because the interests or preferences of the relevant audience are not fixed (and so cannot be explained by the logic of consequences) and because it may not be clear that the behaviour violates what is deemed appropriate, or, more generally, what may be deemed appropriate may not yet be determined (and so cannot be explained by the logic of appropriateness). It may not be clear that the behaviour violates what is deemed appropriate because of the lack of information, for instance, about the situation. Rather, the matter is more complex. On the one hand, the critic is engaging in the logic of consequences: it is using what Risse calls ‘rhetorical action’ in order to realise its fixed but normatively valuable preferences or views, which are not up for negotiation. It does so by persuading *others* in the global public sphere, who are subject to the logic of arguing, that the wrongdoer *should* be subject to a loss of legitimacy — in effect, by the logic of appropriateness. Thus, diplomatic rebuke can be effective since the critic presents an argument in order to persuade the international community of the wrongness of the violation, which can lead to the delegitimation of the wrongdoer.^8^

The efficacy of international criticism by states appears to be supported by the recent quantitative literature on naming and shaming by NGOs, the international media and international organisations. Central pieces in this literature hold that although naming and shaming by these actors might make little difference in redressing lesser human rights violations and could potentially be counterproductive (Hafner-Burton, 2008), it can be important in helping to tackle genocides and politicides (Krain, 2012) and government killing (DeMeritt, 2012). The case presented in this quantitative literature for the efficacy of naming and shaming is somewhat analogous to my defence of the efficacy of diplomatic criticism. For instance, in explaining his findings that naming and shaming works, Matthew Krain (2012) posits that this is because naming and shaming can inform about abuses, frame perpetrators as pariahs, damage their legitimacy, activate powerful bystanders who can impose costs on them and, ultimately, lead to changes in policy.^9^

### Contribution to global norms

Beyond the tackling of the immediate situation in question and the exertion of international pressure on the norm violator, international criticism can play an important role in the establishment and the upholding of morally valuable international norms and laws. By criticising others — and, in particular, by *publicly* criticising others — states may express their view that there are certain norms in the international system and that these have been violated by the aggressor. This helps to reinforce the claim that the norm against the transgression exists, in addition to any action in support of the norm (e.g. economic sanctions) — it makes it clear that the action is in support of the norm. For instance, the US’s strong rebuke of the use of chemical weapons by the Assad regime in Syria and Barack Obama’s assertion that their use would cross a ‘red line’ appeared to strengthen and even advance the norm and laws against the use of such weapons. Subsequently, in September 2013, the UN Security Council passed Resolution 2118, which was lauded by US Secretary of State John Kerry (2013) as ‘declaring together, for the first time, that the use of chemical weapons … [is] also a threat to international peace and security’.^10^

To put the point more precisely, states have prima facie duties to be ‘norm entrepreneurs’ and to be ‘norm carriers’, where relevant. In constructivist IR, actors that develop and propagate new international norms are referred to as ‘norm entrepreneurs’ (Finnemore and Sikkink, 1998: 896). Actors that help to establish further existing norms are ‘norm carriers’ (Heinze, 2009: 124). The aim is to achieve a ‘norm cascade’ whereby the norm becomes adopted by states because of international socialisation (Finnemore and Sikkink, 1998: 902). Once cascaded, norms can then become internalised within the actors so that conformity becomes almost automatic (Finnemore and Sikkink, 1998: 904). International criticism can play a role in norm entrepreneurship and development. When criticising others, states can assert that the practice contravenes what *should* be viewed as a widely held norm. They may then go on to defend their own, more detailed account of the norm, or simply help to cascade existing emerging norms. A potential example of the former is the Brazilian rebuke of the NATO bombing campaign in Libya in 2011 for (allegedly) going beyond the mandate of Resolution 1973 and then its subsequent development of the ‘Responsible while Protecting’ (RwP) concept. In response to the perceived problems of the Libyan campaign, RwP emphasises that those considering undertaking humanitarian intervention need to consider alternative measures first, that interveners need to take extra care when using military force to protect civilians, that interveners should report continually to the UN Security Council and that there are guidelines for humanitarian intervention (for the UN Security Council). Although RwP has not been formally adopted, the import of this contribution in terms of clarifying and developing R2P for future instances of military intervention has been widely noted (e.g. Pattison, 2013; Stuenkel and Tourinho, 2014) and RwP might be seen as Brazil engaging in norm entrepreneurship (Benner, 2013).

The contribution to global norms seems particularly relevant for emerging norms — norms that are not fully established — such as, on most accounts, some of the R2P (specifically, the international remedial responsibility to protect).^11^ However, it may also be relevant for international law insofar as a central element of customary international law is *opinio juris* — the belief among states that certain norms are legally binding. The public expression of criticism may be expressed legally (e.g. the state in question is criticised for having violated international law with their transgression of state sovereignty) and therefore helps to provide an important assertion of *opinio juris*. When the international law is contested, the statement might be influential and help to establish or to uphold the view that a certain morally valuable law *is* law. This may have highly beneficial long-term consequences for the international system.

This point can be taken further. It is not simply that states need to avoid condoning the moral behaviour of other states; they also need to avoid being *complicit* in it. Why is complicity wrong here? After all, complicity can have several forms, with some forms not being morally wrong, all things considered (Lepora and Goodin, 2013). My claim is this: when states fail to register rebuke of those that engage in internal or external aggression, they may be complicit in future situations of aggression (as well as of the current situation). The complicity may stem from the fact that their silence can be reasonably foreseen to be perceived by other parties, including the aggressor, to be an act of condoning the continuation of the aggression under consideration or of future aggression of a similar nature.^12^

### Punishment

Third, those who engage in egregious wrongdoing should be punished. The international system is, however, fairly devoid of means of punishment. International criminal tribunals and the International Criminal Court (ICC) provide means of punishment for *some* of those engaged in external aggression and mass atrocities. However, the powers of the ICC and such tribunals are very limited and their shortfalls are well-known; I will not restate them here.

This does not mean, however, that there is *no* punishment available in the international system. Certain forms of targeted economic sanctions, such as asset freezes and travel bans, can punish leaders responsible for egregious crimes. Similarly, diplomatic criticism, such as in the form of widespread opprobrium, can punish decision-makers (who should be the focus of punishment) since they face reputational loss.^13^ The reputational loss that stems from such sanctions may not be *all* that such leaders deserve — they might deserve, for instance, life imprisonment. Notwithstanding, it seems that such leaders deserve *at least* international opprobrium and the accompanying reputational loss. Consider, for instance, the general tarnishing of the reputations of Tony Blair and George W. Bush after their War on Terror and War in Iraq. This was established by the widespread critique of their policies and (it seems to me) was the least they deserved. Furthermore, even if leaders *are* subject to other forms of punishment, such as targeted economic sanctions, they may deserve reputational loss *as well*. To be sure, decision-makers may suffer a loss of legitimacy simply by the tacit rejection of their policies in the global public sphere. However, it seems that this is more likely with the public, explicit rejection of their policies, which can come with a clear public denouncement of their actions.

## The alternatives

I have thus far presented three reasons in favour of engaging in diplomatic criticism. In order to establish that there is a prima facie duty to often criticise and so to often engage in diplomatic criticism instead of the alternatives (e.g. according to the comparative conditions of Just War Theory, such as last resort), we need now to consider some of the problems of the alternatives. I will first compare diplomatic criticism to war and economic sanctions, before comparing it to other diplomatic measures and, in particular, dialogue, mediation and the cutting and downgrading of diplomatic ties.

### Comparison to war and economic sanctions

There is a straightforward problem with war and economic sanctions that I will not belabour here: although diplomatic criticism may sometimes cause *some* harm to innocents (e.g. if the criticism delegitimises the state and, in turn, harms its trade, leading to economic hardships), the magnitude and likelihood of harm to innocents on both sides of war and economic sanctions will typically be much larger. The internal costs to the warring or sanctioning party are also likely to be much greater than if they were engaged in only diplomatic criticism. To that extent, war and economic sanctions are more likely to be worse under the comparative conditions of Just War Theory, such as necessity and last resort.^14^

Perhaps less noted is a related point that war (and, to a lesser extent, economic sanctions) are problematic since only a few states may permissibly launch a war (and, to a lesser extent, economic sanctions) given the costs involved. Wars and interventions can generally be undertaken effectively only by states with significant military capabilities. For instance, in most (but not all) cases of humanitarian intervention, states would require hugely expensive airlift or sealift capacity in order to intervene in other states.^15^ Accordingly, in response to many cases of external aggression and mass atrocities, there are very few states (and sometimes no states) that could wage war effectively, by doing sufficient good to outweigh the harms of the use of force. Given that Just War Theory requires that action be likely to be effective under a principle of proportionality or the principle of a reasonable prospect of success (depending on the particular account of *jus ad bellum* held), it would follow that most states should not wage war, even if war would be the last resort, because of the likely disproportionate costs of war.

By contrast, diplomatic criticism can be pursued not simply by the potentially warring parties as an alternative or addition to war or economic sanctions, but also by less materially capable states that do not have war or economic sanctions as a feasible option. Since the costs to innocents are generally much lower with diplomatic criticism, the launching of diplomatic criticism need not be likely to achieve as much good in order to outweigh such harm, compared to war or economic sanctions. Diplomatic criticism also seems to be a measure that less materially powerful states can use to exert influence in a manner that is much greater than their material capabilities. This is because they may have the better arguments and so influence international public opinion to exert pressure on the wrongdoers in a manner that exceeds their capabilities, and potentially have much more influence than more militarily powerful actors whose hypocrisy or entrenched interests may remove some of the influence of their critique. Accordingly, diplomatic criticism is preferable since it imposes lower costs on innocents and because it is likely to be a permissible option for many more states.

### Comparison to other diplomatic measures

#### Dialogue

It might be thought that rather than engaging in diplomatic criticism, states should instead engage in dialogue with those involved in external aggression and mass atrocities. This is because diplomatic criticism may be viewed as preaching or ‘monologue’ — or, more colloquially, ‘megaphone diplomacy’ — and so be unlikely to be effective. Indeed, the central premises of the notion of ‘public diplomacy’ are relationship-building and dialogue, which are claimed to be more likely to achieve the broader, long-term aims of diplomacy.^16^ The sharing of understandings and listening to others’ complaints and viewpoints, the argument goes, is much more likely to lead to a shift in behaviour than the strong rebuke of offending agents. For instance, criticising China’s human rights record might seem unlikely to lead to a shift in policy; engagement and long-term dialogue might be viewed as what is required to lead to such a shift. Monologue may be viewed as moralising, perhaps leading to the entrenchment and potentially illicit but influential accusations of hypocrisy. By contrast, by giving all the parties a voice, dialogue might offer opportunities for engagement and, in turn, increased positivity towards opposing viewpoints and the potential acceptance of political outcomes as fair (Cowan and Arsenault, 2008: 19). This may not only tackle the immediate problems of internal or external aggression, but also lead to a shift in approach of the actors so that they do not engage in such behaviour in the future. In a somewhat similar vein, Paul Sharp (2009: 10–12), in his account of a ‘Diplomatic Theory’ of IR, argues that ‘thinking diplomatically’ privileges the maintenance of relations and that ‘diplomatic understanding’ suggests ‘quietism’. He notes that one of the implications of the Diplomatic Theory of IR is ‘the morality of suspending judgement’ (Sharp, 2009: 299). This, he suggests, stems not only from the import of continued dialogue and retaining ongoing relations, but also from moral scepticism stemming from a pluralistic international society and the resultant epistemic uncertainty about purported moral universals, such as those that give rise to calls for humanitarian intervention.^17^

In addition, it might be claimed that dialogue is intrinsically valuable. Thus, some accounts of public diplomacy assert that states should not simply engage in relationship-building and dialogue with other states in order to achieve the foreign policy goals of the state. Rather, one should understand the case for public diplomacy as depending, in part, on the intrinsic value of listening to and debating the issues — in short, the interaction (e.g. Cowan and Arsenault, 2008: 19; Fitzpatrick, 2007: 208).

How strong is the case for dialogue over monologue? It seems clear that dialogue may *sometimes* be better than preaching. Sometimes, states should not engage in public, international criticism. As such, the duty to engage in such criticism is only *prima facie* (i.e. it applies only to certain cases) and *pro tanto* (i.e. the non-instrumental and instrumental reasons for it may be outweighed by the reasons against). However, an *outright* rejection of monologue would be mistaken.

First, the case for monologue is stronger than is often perceived. We should judge monologue not simply by the efficacy of tackling the immediate situation, but also by the other considerations in favour of criticism discussed earlier. For instance, if the argument discussed earlier about the contribution to international norms is correct, there is a longer-term, countervailing consideration in favour of such criticism. This is often missed with the defences of dialogue. That is, the aim of diplomacy should not be simply to persuade the state in question from refraining from their abusive action — which is the goal of dialogue — but also to persuade *other* states to refrain from doing so.

Second, although dialogue may sometimes succeed, we should be realistic about its success and the comparative success of monologue. In many cases, both will fail to tackle the immediate crisis or longer-term abuses. For instance, China might not listen to the proclamations of certain states about its human rights record. Yet, it also seems unlikely that dialogue on this issue will achieve much success either. In this context, Katrin Kinzelbach (2014: 2) argues that the secretive European Union (EU)–China Human Rights Dialogue has delivered very little; there remains continued disagreement and there has been very little long-term influence on China’s compliance with human rights. On the contrary, the EU–China Human Rights Dialogue has, according to Kinzelbach (2014: 197), diluted international pressure on human rights, relegating and isolating human rights concerns from high-level political discussion. Dialogue is therefore no panacea and the likelihood of success should be judged accurately, rather than holding false hope of agreement.

Third, the independent value of dialogue at the international level seems weak. To return to Risse’s account of communicative action discussed earlier, dialogue would involve both the critic and the wrongdoer negotiating about the alleged serious external aggression or mass atrocities. The preferences and interests of both actors would not be fixed: both actors would be open to be persuaded. The worry, however, is that in the sorts of cases that this article is primarily focused on — external aggression or mass atrocities — it would not be *morally desirable* for the critic to be persuaded that external aggression and/or mass atrocities are justifiable. A reasoned consensus that reaches this conclusion would be morally bankrupt. In such cases, dialogue may only be appropriate, then, for the empirical question of establishing the facts of the case, but even then, the evidence that the critic possesses may be incontrovertible and so they should *not* be persuaded. Dialogue may seem more valuable when the normative case is ambiguous; reasoned consensus may then be of value. However, any value of such a consensus will not stem from the process of dialogue *itself*. As Risse (2000) notes, dialogue in the international system is far from the Habermasian Ideal Speech situation given, for instance, entrenched inequalities. As such, it seems implausible to hold that international dialogue is intrinsically valuable, even if the better argument does win.

Fourth, and perhaps most important, dialogue and the duty to criticise are not necessarily incompatible. Rather, as some advocates of public diplomacy (e.g. Cowan and Arsenault, 2008; Gilboa, 2008) note, they are appropriate for different contexts. In the longer term, dialogue can build trust and friendly relations that can be used to persuade states to shift their outlook and not to engage in internal or external aggression. In the short term, when the internal or external aggression is impending or ongoing, it seems that monologue will often be preferable since there is not the time required for relationship-building and persuasion. In addition, dialogue and monologue may run continuously so that the state both publicly repudiates and tries to persuade privately at the diplomatic level. Furthermore, on occasion, dialogue may have been tried and have failed, and so it becomes necessary to resort to monologue. To that extent, dialogue can often be seen as a plausible option of ‘first resort’ (see below), that is, a (longer-term) option to be tried before resorting to war or other more coercive options. This is still consistent with holding that diplomatic criticism should also be launched on occasion before more coercive options, and even sometimes instead of dialogue, when dialogue is unlikely to be successful.

#### Mediation

It might also be claimed that mediation (by a third party) should be tried before monologue. Mediation involves the persuasion of the parties by listening to grievances and explaining how interests and identities may be realised by changing the behaviour to avoid the impending action, as well as, on occasion, the offering of incentives and the threat of political (and other) sanctions. Conducting diplomacy quietly through mediation gives states a means of changing their behaviour without losing public face. Mediators, Berridge (2010: 236) argues, need to be impartial in the dispute. Being a public critic of one of the parties would seem to potentially undermine this impartiality.

Yet, in similar vein to the point made earlier about dialogue and monologue, mediation and the duty to criticise may be compatible. Where appropriate, it may be that mediators should *threaten* to criticise publicly first (along with other potential incentives and sanctions). This gives the offending state(s) a chance to redress their behaviour before they face the sanction. Mediation can, then, be seen as a first resort, before resorting to public criticism. It is generally part of the toolkit of preventive diplomacy, to be considered before crises develop (e.g. the mediation efforts in Kenya in 2008 after post-electoral violence). To that extent, it might be viewed, like dialogue, as a plausible option of first resort in Just War Theory — or, more broadly, in the ethics of conflict prevention — to generally (if not always) be tried before resorting to war or other more coercive options (see below).

As such, and to reiterate, my aim in this section is not to repudiate the case for mediation or dialogue. To be clear, I think that there are often good reasons for pursuing these options. Rather, my point is that the case for them does not repudiate the case for the prima facie duty to criticise because diplomatic criticism and mediation or dialogue may be compatible and because dialogue also faces some problems.

#### The cutting and downgrading of diplomatic ties

Let us now consider the case for the significant downgrading and severance of diplomatic ties with the state engaged in mass atrocities or external aggression. There are, on the face of it, two immediate, potential attractions of cutting and downgrading diplomatic relations. First, by distancing oneself from the offending state, states can make it clear that they view the wrongdoing as illegitimate. This could seemingly help in furthering some of the central reasons to engage in diplomatic criticism: it might be thought to increase pressure on the wrongdoers by delegitimising them and to make it clear that the wrongdoing contravenes morally important international norms. Second, and related, cutting ties may be thought to remove any potential complicity in the wrongdoing. The sanctioning state cannot be said to condone or enable the wrongdoing. By contrast, if states maintain their presence within aggressors (and vice versa), the wrongdoing may be perceived to be condoned. To the extent that such presence provides aggressors with a veneer of legitimacy, those who maintain such presence may be viewed as enabling the maintenance of the wrongdoing (which withdrawal would redress) and therefore as complicit in it.

However, there are notable countervailing worries with the cutting and downgrading of ties, which do not apply to the same extent to diplomatic criticism.^18^ On the one hand, diplomatic criticism *already* makes it clear that the wrongdoer should be delegitimised and norms that are morally important have been violated. The cutting and downgrading of ties seem likely to contribute little in this regard. Indeed, in his recent quantitative study, Matthew Krain (2014: 46–47) finds that these measures do little to make things better ‘and under particular conditions may even make things worse’. In similar vein to my point here, Krain posits that diplomatic disengagement fails to work because it does little to raise the costs for wrongdoers beyond the implicit threats and the delegitimation that already comes from naming and shaming.

On the other hand, the cutting and downgrading of ties may be counterproductive in terms of addressing the immediate wrongdoing. The withdrawal of ambassadors and the cutting of diplomatic ties can lead to a loss of intelligence and could reduce the ability of the sanctioning state to communicate to the aggressor the complaint that they have and to make it clear what needs to be done in order to redress or prevent further wrongdoing. This is because one of the primary roles of an embassy is to communicate between the sender and the target state in order to explain decisions and to forge relationships (Maller, 2010: 66; see also Berridge, 2010: 103–124). For instance, Tara Maller (2009) argues that the US’s cutting and downgrading of ties in Afghanistan and Sudan in 1989 was counterproductive for its counterterrorism policy. In the case of Afghanistan, the decision to cut democratic ties weakened the US’s ability to make accurate decisions in response to the rise of Osama bin Laden and the Taliban, and missed the chance to strengthen the moderate sectors of Afghani society (Maller, 2009: 517–525). In the case of Sudan, diplomatic disengagement led to a lack of sufficient reliable intelligence on Sudan and ultimately to the misguided Al Shifa strikes that strengthened anti-US feeling among radicals (Maller, 2009: 525–529).

Moreover, the cutting and downgrading of ties may also have longer-term implications for the relations between agents, making it harder to achieve changes in the wrongdoer to avoid future instances of mass atrocities and serious external aggression. In other words, diplomatic disengagement can undermine the potential for dialogue, which, as noted earlier, may be beneficial in the long term. As Maller (2009: 513) argues, diplomatic ties provide a means by which a state can engage in public diplomacy to project a positive image of the state abroad and to provide channels of communication to work out differences and to resolve conflicts before they escalate. Continued engagement is required for a clear articulation of interests, as well as to enable parties to humanise each other, to build trust and to foster greater understanding (Maller, 2009: 530).

In sum, the cutting and downgrading of political ties should generally (if not always) be eschewed. By contrast, dialogue and mediation may sometimes be appropriate, but there is still sometimes a duty to engage in international criticism in cases of external aggression and mass atrocities. Indeed, this duty will apply in more cases than it may first appear since the instrumental case for dialogue also faces problems, the non-instrumental case for dialogue seems weak and monologue may be compatible with dialogue and mediation.

## Objections

Thus far, I have presented the case for international criticism as an alternative and addition to other means of addressing mass atrocities and external aggression. I will now consider two potential responses.

### Response one: The Demandingness Objection

The first response is that the duty to criticise is extremely demanding. That is, it requires of states and other agents too much. By engaging in such international criticism, states may, for instance, lose important trade deals. The objection, then, is that the duty to criticise assumes a particularly demanding form of cosmopolitanism, which asserts that individuals and states have very strong duties beyond their borders. As such, even if diplomatic criticism will address the situation, and be the best response under comparative Just War conditions such as last resort, it would not be permissible. This is because it would be disproportionate with regard to the internal costs borne by states. It would be worse than doing nothing given the costs for the population of the state criticising.

The worry about demandingness can, however, be largely defused by making two related points. First, as noted earlier, diplomatic criticism is often much less costly than other measures to address mass atrocities and serious external aggression. Second, common-sense morality appears to hold that the costs that states may be asked to bear in terms of the tackling of egregious human rights violations may be quite high. It is widely held that, in certain cases, states have a moral duty to undertake military intervention in order to tackle egregious violations of human rights (see, generally, ICISS, 2001). This is despite the fact that the financial costs of intervention are often very significant (e.g. the costs of the Kosovo intervention, including post-war reconstruction, is estimated at USD46 billion in the ICISS (2001: 71) report on R2P). This is also despite the fact that there may be casualties when doing so (although in certain states, such as the US, there seems to be less of an acceptance of casualties). Hence, *if* we accept that efficacious military intervention to respond to mass atrocities may be morally required, despite its costs, it would seem that efficacious diplomatic criticism that could similarly tackle mass atrocities might also be morally required, despite its costs.

Moreover, when responding to aggression against one’s own state, citizens may be willing to accept even greater burdens than that which they can be required to bear in the tackling of mass atrocities. This may mean that it is permissible for states to take on even greater costs when engaging in self-defensive measures. The costs of forthright criticism in response to external aggression therefore seem unlikely to exceed the level of costs that states can permissibly take on in response to external aggression. Accordingly, even seemingly high internal costs would not rule out diplomatic criticism as a disproportionate option.

### Response two: The Hypocrisy Objection

The second objection to the duty to criticise is that states are not entitled to criticise others in many cases. This is, in particular, when they are being hypocritical. That is, when states have previously engaged in the same or a similar practice, they lose their entitlement to criticise others for engaging in that practice. The objection about hypocrisy is one of the most common objections levelled against those who engage in diplomatic criticism. It is used by those engaging in morally problematic behaviour to deny that the agent criticising them has the right to do so. For instance, in response to US criticism of its human rights record, in 2012, the Russian Foreign Ministry issued a 50-page official report cataloguing US violations of human rights, including rendition, police discrimination and drone strikes (Weir, 2012).

The scope of this objection may appear to be very broad. Nearly all states in the international system face *some* human rights problems internally or have previously engaged in or supported aggressive action. For instance, the coalition for the (in my view, aggressive) 2003 War in Iraq was made up of 40 states, roughly one in five of the states in the world. More broadly still, the Amnesty International (2013) annual report discusses human rights issues in over 160 states.

Why is hypocrisy wrong? Most fundamentally, at the interpersonal level, hypocrisy seems wrong because, as R. Jay Wallace (2010) argues, it appears to deny the equal moral standing of other agents. At the international level, the worry is very similar. When a state criticises another but engages in the same practice itself, it is effectively asserting that the same standards do not apply to *it*. The perhaps implicit claim is that it is exceptional — and superior — to other states. Hypocritical criticism also seems problematic instrumentally since it can weaken the impact of the hypocrite’s (even correct) critique since the critique is perceived to lack credibility (Mor, 2012: 394). The critique, when the hypocrisy is public, may be regarded with suspicion and dismissed. In addition, hypocrisy may be problematic because of its effects on the international community. It can undermine the practice of an appeal to international norms by means of, for instance, diplomatic criticism since such appeals are viewed cynically and sceptically, especially with regard to the motives of the state that is making the criticism. The state may not take seriously the requirements of the norm itself and may not believe that, regardless of its rhetoric, conformity is, in fact, morally or legally required by it. To that extent, hypocrisy is a worry because it can undermine the status of norms as requiring conformity. It appears to show that the norms or laws governing international society are, in fact, weak or non-existent since they do not compel action for all.

Nevertheless, it seems that the force of the Hypocrisy Objection is severely limited. To start with, although hypocrisy has some independent moral wrongness, this seems of *small* moral concern, all things considered. This is because it is attitudinal in that it concerns the attitudes that we hold about others’ moral standing — the mindset of being superior to others. Such mindsets seem generally of little independent moral import when it comes to considering matters of international politics. For instance, the independent moral import of an agent’s motives seems small when considering the much larger harms involved in wars and mass atrocities in Just War Theory (see Pattison, 2010: 156–161; 2014: 26–36). Indeed, much of the intuitive force of what seems to be the independent moral wrongness of hypocrisy concerns not the hypocrisy *in itself*, but rather the *substantive* failure to meet the requirements of morality, such as by wrongfully supporting dictators or by invading other states. For instance, critics of US hypocrisy often attempt to expose substantially bad acts or omissions (Glaser, 2006: 263), such as its violation of rights in Guantanamo Bay or failure to intervene in Rwanda. It is not the inconsistency *per se* that is wrong here, but rather the substantive failure to do what should be done. We need, then, to consider cases where our judgements about the independent moral wrongness of hypocrisy are not affected by substantive wrongs. So, let us consider a case of consistent, but morally problematic, treatment. Suppose that the UK ignores mass atrocities in Bahrain but then criticises both mass atrocities in Syria and others’ failure to respond to the Syrian situation. It should, of course, criticise in both cases. However, if it has *already* failed to criticise Bahrain, it seems better that it be hypocritical and still criticise mass atrocities in Syria (and others’ failure to respond) than not to, even on pain of hypocrisy. Although failing to criticise in the case of Syria would be treating both sets of individuals equally, it seems that states should treat *some* well rather than *all* equally badly. As such, states can permissibly — and perhaps should — criticise, even on pain of hypocrisy, when doing so asserts that a particular group should not be subject to mass atrocities.

In addition, hypocritical criticism may sometimes still be efficacious. First, the criticism may play an educative role in helping to establish facts about the case unbeknown to other agents. Second, the criticism may add to the cacophony of voices that condemn the action and the hypocrisy may have little negative instrumental impact on international norms. Third, if hypocrisy is the homage that vice pays to virtue, then hypocrisy can, on occasion, help to reinforce norms. This is particularly so when the hypocrisy is unexposed (Glaser, 2006: 262). Fourth, in asserting and potentially strengthening the norm, hypocritical criticism provides a means by which others can criticise the hypocrite when the hypocrisy is exposed. In short, they should practise what they preach. Indeed, hypocritical states may be affected by their hypocritical justifications for action and amend their behaviour accordingly in order to be more virtuous, in large part due to concerns about their reputation (see Goodman, 2006). To be clear, these considerations may not fully repudiate the negative consequences of hypocrisy. Nevertheless, they show that some of the instrumental worries surrounding hypocrisy may not always be serious.

Moreover, although the scope of the Hypocrisy Objection is, at first sight, broad, it is, in fact, quite small. Jerry Cohen (2013: 134), in discussion of the related issue of ‘*tu quoque*’ (‘you too’) critiques, notes that ‘[f]ew of us think that no one can call the kettle black who has committed sin of *any* kind’. He considers two alternative views (Cohen, 2013: 135–137). On the ‘quantity view’, it is appropriate to criticise only when your sin is less than the sin committed by those whom you are criticising. On the ‘quality view’, it is appropriate to criticise only when your sin is of a different kind to the sin committed by those whom you are criticising. Like Cohen (2013: 140), I think a combined account, whereby the sin needs to be of the same kind and seriousness, is the most intuitively plausible account of hypocrisy. On this combined account, when the sin is of a different kind (e.g. not killing, but other, less serious rights violations, such as denials of freedom of expression) or when the sin is of a lesser quantity (e.g. such as killing one in a bundled, mendacious police operation, but not mass killing), the state is not acting hypocritically when they criticise others.^19^ Moreover, it seems that the agents accused of hypocrisy (or other lapses) still need to be committed to or engaged in their wrongful action. If they subsequently denounce and apologise for their wrongful action — that is, if they show that they no longer have the problematic mindset — it does not seem that they can be rightly rebuked for being hypocritical since they no longer deny others’ equal moral standing.

In sum, states should not engage in hypocritical international criticism. They should act rightly — and not engage in internal or external aggression themselves — and possess the attitude that others are morally equal. However, their failure to do either — to act rightly or to have the right attitude — does not foreclose the possibility of permissible diplomatic criticism that is hypocritical. It will often be preferable that they engage in diplomatic criticism despite the apparent hypocrisy; alternatively, the criticism will not, in fact, be hypocritical.

## Diplomacy and last resort

I will now consider how my case for diplomatic criticism is relevant for accounts of Just War Theory and, in particular, the principle of last resort.^20^ I will distinguish between three accounts of last resort in the existing literature in Just War Theory, drawing on a recent, important critique of this principle by Eamon Aloyo (2014).^21^ The first account is (as referred to in the Introduction) the ‘Literal Account’, which requires that *all* peaceful options be tried before war is permissible. One problem with the Literal Account, as famously noted by Michael Walzer (2004: 88), is that ‘[t]here is always something else to do: another diplomatic note, another United Nations resolution, another meeting’. War is therefore *never* an option. In addition, the Literal Account seems problematic since it holds that even if the other options — such as mediation and the cutting of ties — are unlikely to succeed, and even if they will be counterproductive in addressing the situation, they should still be tried.

The second account is the ‘Reasonable Chance Account’. This is a non-literal account: it holds that only peaceful options that have a reasonable chance of success should be tried first. This improves upon the Literal Account since if, for instance, dialogue will not have a reasonable chance of success, then it would not be required to be pursued before war is launched. Yet, the problem with the Reasonable Chance Account is that the options that *do* have a reasonable chance of success may still be disproportionate, indiscriminate and unnecessary, yet required to be tried (Aloyo, 2014: 4–6). For instance, if economic sanctions would have a reasonable chance of success at tackling mass atrocities but would be likely to lead to far more deaths of innocents than a targeted aerial-bombing campaign, sanctions would still have to be pursued before war would be permissible. The problem, then, with the Reasonable Chance Account is that it requires insufficient comparison of the costs and benefits of the various options.

The third understanding of last resort holds that the resort to war is permissible only if it will cause the least amount of harm. Following Simon Caney (2005: 202, 249), we can call this the ‘Least Awful Account’. Unlike the Reasonable Chance Account, this account of last resort is sufficiently comparative: it requires that the costs and benefits of the various options be weighed and the least awful option be chosen. War may be undertaken ahead of economic sanctions or various diplomatic measures, such as diplomatic criticism, if it is the least awful option. However, Aloyo (2014: 6) argues that such an account is ‘specious’. This is because, he claims, it is not really a principle of last resort; it fails to indicate why war *in particular* should only be attempted after the other options have been tried first given that war may be better than the other options. In other words, it loses any notion of ‘lastness’ in the last resort principle. In addition, he argues that, on this account, the last resort principle is redundant because it is already covered by other Just War principles, most notably, necessity (or an account of proportionality (e.g. Hurka, 2005) that includes comparative concerns).

Given the problems with these three accounts, Aloyo (2014: 6) argues that the last resort principle should be dropped ‘as a just war theory requirement’. This is an important challenge, but there is a fourth, more plausible account of last resort, which the analysis earlier of the case for diplomatic criticism helps to illuminate. This is an amended version of the Reasonable Chance Account and the Least Awful Account, which I will call ‘Presumptive Last Resort’. Presumptive Last Resort holds that: ‘*(i) war should (generally) be the last feasible option; and (ii) the comparatively best non-violent, feasible option(s) should be tried first*’.

Underlying Presumptive Last Resort is a comparative principle that requires a weighing of the various feasible options at addressing the situation. To that extent, the underlying basis of this principle is akin to an *ad bellum* principle of necessity, where necessity is understood to require, in short, the option — whether violent or non-violent — that will be expected to do more good than harm overall. The weighing of the various goods in such a calculation should not be viewed in narrow instrumentalist terms whereby they are each assessed simply according to their likely efficacy at addressing the situation and some of their costs. The preceding analysis of the case for diplomatic criticism has made clear that the case for this option, for instance, is not simply narrowly instrumentalist. Rather, it concerns a variety of factors, including the (small) independent moral import of hypocrisy, the potential independent value of punishment and the contribution to global norms.

Another important consideration that should be included in the underlying calculation concerns the difference between *doing* and *allowing* harm, which is widely held in the literature on moral philosophy to have *some*, but not absolute, moral significance.^22^ That is, it is *somewhat* morally worse to *do* harm oneself (e.g. if a humanitarian intervener unintentionally but foreseeably kills civilians in a bombing raid), even if this *allows* for the same or more harm overall (e.g. if more civilians will die without humanitarian intervention). Underlying Presumptive Last Resort, therefore, is an account of necessity that compares the various military and non-military options, weighing the various goods and harms, and, in doing so, holds that *doing* harm should generally be avoided.^23^ From this basis, Presumptive Last Resort requires that: (i) war should (generally) be the last feasible option; and (ii) the comparatively best non-violent option(s) should be tried first.

What grounds the presumption in clause (i) that war is generally likely to be comparatively worse than the other options? First, it seems that, despite some potential exceptions, war will generally be likely to be worse in narrow instrumentalist terms given the death and destruction that it brings. This is, I think, reflected in Walzer’s (2004: 88) claims that:

sending troops into battle commonly brings with it so many unanticipated costs that it has come to represent a moral threshold: political leaders must cross this threshold only with great reluctance and trepidation. This is the truth contained in the ‘last resort’ maxim.

Second, beyond narrow instrumentalist calculations, the other options, such as diplomatic criticism, seem less likely to involve as much *doing* of harm as war, even if they may *allow* for more harm overall (e.g. if war would sometimes be more effective at addressing the situation).

To reiterate, Presumptive Last Resort, as its name suggests, maintains a *presumption* — but not an *absolute prohibition* — against resorting to war first and in favour of pursuing other options instead. Sometimes, the presumption will *not apply* since it will be very clear that war will *do* less harm and be more effective than the other options, notably, such as economic sanctions (and be favourable with regard to the other normative considerations). In addition, even when war will *do* more harm than the other options, it may sometimes be clear that the presumption can be justifiably *overridden* given that the difference between doing and allowing is not absolute. For instance, although it may *do* more harm, it may on occasion be clear that war may be comparatively a *much better* option in narrow instrumentalist terms — for example, it will be by far the most effective measure to address ongoing mass atrocities. The import of achieving such beneficial consequences may be so great that it outweighs the other considerations against going to war (see, further, Pattison, 2010).

Although the underlying basis of this account of last resort can be captured by other moral principles, such as necessity, this does not render the principle redundant. Importantly, last resort contains a contingent, non-absolute presumption against war. This is practically important to include in Just War Theory in order to emphasise the wrongness of war and the case for pursuing the alternatives. In short, war may *sometimes* be permissible and other options may not, but war should *generally* be avoided and the other options should be pursued. Last resort helps to emphasise this.^24^ In addition, although the underlying basis of Presumptive Last Resort may be captured by a nuanced principle of necessity or a comparative account of proportionality, this does not mean that we should forgo last resort. Rather, it seems that we should drop necessity from *jus ad bellum*. This would cohere with much of the tradition of Just War Theory: unlike last resort, necessity is rarely included in the principles of *jus ad bellum* (although it is included as an *in bello* principle).^25^ Furthermore, importantly, Presumptive Last Resort, unlike necessity, contains a presumption against going to war.^26^

Let us now consider the second part of Presumptive Last Resort, which is that (ii) the comparatively best non-violent option(s) should be tried first. This goes beyond traditional accounts of last resort to offer an account of first resort, second resort, third resort and so on, in similar vein to the Least Awful Account. This may go beyond *jus ad bellum* per se, which is, of course, focused on the ethics of launching *war*. However, it is clearly important more generally for the ethics of foreign policy and for addressing mass atrocities under R2P and serious external aggression.

There is not a strong presumption about which option should be favoured, unlike the presumption against war, although we have seen that dialogue and mediation could potentially be options of first resort, with diplomatic criticism perhaps to follow as a second resort. A more precise account of the ranking of the alternatives is beyond the scope of this article given that this would require an assessment of the ethics of all the various options in sufficient detail (e.g. economic sanctions, diplomacy, financial inducements, international criminal tribunals and arming rebels). Yet, if we are to have a persuasive and complete response to the issue of how states and other actors should respond to aggression and mass atrocities — and when war can be just — we cannot ignore the ethics of these alternatives to war. By offering an account of some of the normative considerations raised by diplomatic criticism, this article is, I hope, a helpful starting point in this exercise.

## Conclusion

I have, then, defended the case for diplomatic criticism in response to external aggression and mass atrocities. States have a prima facie duty to criticise offenders in order to tackle the situation at hand, to contribute to the relevant global norms and to punish. The practical recommendations that follow from this analysis are clear: states and other actors should publicly denounce external aggression and mass atrocities much more than they do. To that extent, diplomatic criticism is a central means of helping to fulfil the R2P. Diplomatic criticism should often be undertaken (1) instead of war and economic sanctions and other diplomatic measures. It should also often be undertaken instead of (2) doing nothing. Furthermore, even when war, economic sanctions and other diplomatic measures are to be undertaken, diplomatic criticism should often be undertaken (3) as well. To that extent, it is often a plausible and desirable option under the account of the Presumptive Last Resort principle that I have delineated.

## Notes

1. These accounts are discussed further in the penultimate section.

2. For analysis of the ethical issues raised by sanctions, see Pattison (forthcoming).

3. On the R2P, see, for instance, ICISS (2001) and Ban (2012).

4. As far as I am aware, the only exceptions are Bjola (2014), Bjola and Kornprobst (2013), Gross (2013) and Toscano (2001).

5. In similar vein to what I will argue, in August 2014, Ed Miliband, the leader of the UK Labour Party, criticised David Cameron for his ‘inexplicable’ silence on ‘the killing of hundreds of innocent Palestinians caused by Israel’s military action’ in Gaza (Boffay, 2014).

6. Criticism is also important for getting the situation onto the international agenda (Risse, 2000: 20), such as mass atrocities that have not been previously discussed by the UN Security Council.

7. See, for instance, the cases of Indonesia and Morocco discussed by Risse (2000: 29–30).

8. An upshot of this argument is that the duty to criticise should often be *public*: the critic needs to denounce publicly the wrongness of the violation in order to persuade the international community that the wrongdoer *should* be rebuked. Quiet diplomacy will not achieve this.

9. To be sure, the focus of this literature is on naming and shaming by NGOs, the international media and international organisations. There is not, as far as I am aware, a quantitative study of the impact of naming and shaming *by states in general* (the focus of this article). (Franklin (2008) does consider the impact of criticism by states (and other actors) of human rights abuses, finding that it reduces repression, but he focuses on the Latin American case.)

10. Of course, the subsequent failure to intervene militarily in response to the crossing of the red line may be claimed to have weakened the taboo against using chemical weapons. On the other hand, the renewed diplomatic effort and attempted destruction of the weapons may show that the norm violation did, in fact, motivate a clear, robust international response.

11. The R2P involves the international remedial responsibility to protect, which is an emerging norm, as well as norms concerning Pillar One (the protection responsibilities of states) that are well established, for example, in international law.

12. Beversluis (1989) presents a similar argument in the context of economic sanctions.

13. Note that I focus here on the retributivist case for punishment.

14. I will consider this point further and how these principles should be understood in the final section. Note that this problem may not apply to targeted economic sanctions.

15. There are exceptions. For instance, states may be able to intervene much more easily in neighbours, they could provide front-line troops and be supported logistically by other states that do have sufficient airlift and sealift capacity, or they could engage in other military activities, such as supplying arms to rebel groups.

16. I will not offer a definition of public diplomacy here since there is very little agreement in the literature on the topic as to exactly what it involves, bar the need for relationship-building and dialogue. Influential accounts of public diplomacy include Cowan and Arsenault (2008), Cull (2008), Fitzpatrick (2007), Gilboa (2008) and Nye (2008). Interestingly, Berridge (2010: 179–182) argues that public diplomacy is, in effect, simply propaganda. To that extent, it may be viewed as more similar to monologue than the dialogue emphasised by other scholars of public diplomacy (for Berridge, public diplomacy is aimed at the population of the target state, rather than the international community more generally, which, as noted earlier, is the main audience of diplomatic criticism). By contrast, Mellison (2013: 440) claims that the main difference between propaganda and public diplomacy is that the former is generally uninterested in relationship-building and dialogue (see also Sharp, 2009: 270–275).

17. To be sure, Sharp (e.g. 2009: 308) is generally at pains to point out that the moral claims of the Diplomatic Theory of IR may be disputed.

18. Diplomatic criticism need not, of course, end diplomatic relations; it is, by contrast, a particular form of diplomatic communication. See, further, the discussion of the different types of diplomatic correspondence in Barston (2014: 288–348).

19. Criticism of a different scale or kind may, like hypocrisy, also deny the equal moral standing of other agents. However, it would not be hypocrisy per se; it would be a different sort of moral failing (see, further, Wallace, 2010: 336).

20. It should be noted that one may accept my account of the prima facie duty to criticise without necessarily accepting my account of last resort. Conversely, one may also accept my account of last resort without accepting the prima facie duty to criticise.

21. Aloyo lists four accounts, but it is unclear that the second account that he identifies — ‘necessity last resort’ — differs from the other three or is widely endorsed in the literature.

22. Influential accounts of the moral import of the difference between doing and allowing include Quinn (1989) and Scheffler (2004).

23. For a more in-depth account of necessity than is possible here, see Lazar (2012).

24. See, further, Brunstetter (2014), who highlights the rhetorical import of the last resort principle in US presidential debates.

25. It would also seem more straightforward to have a separate principle of proportionality that compares the option under consideration to only doing nothing (rather than comparing all the various options).

26. This account of Presumptive Last Resort has, I think, *some* affinity with the account of last resort presented by John Lango (2014). Lango provides a detailed, nuanced and largely novel account of defence of the moral import of last resort, which he fleshes out with five ‘reasonableness’ standards. Space precludes fully considering his account here (which spans much of the length of his book on Just War Theory), and the similarities and differences to my account. It is worth noting, though, that, like my account of Presumptive Last Resort, he does not include a further principle of necessity and he defends the importance of last resort as deriving, in part, from the import of what he calls the ‘non-harm’ principle (which seems to have some affinity to the difference between doing and allowing).
